# Metabolism of pyrene through phthalic acid pathway by enriched bacterial consortium composed of *Pseudomonas*, *Burkholderia*, and *Rhodococcus* (PBR)

**DOI:** 10.1007/s13205-017-0598-8

**Published:** 2017-04-11

**Authors:** Sagar Vaidya, Kunal Jain, Datta Madamwar

**Affiliations:** Environmental Genomics and Proteomics Lab, UGC Centre of Advanced Study, P. G. Department of Biosciences, Sardar Patel University, Satellite Campus, Vadtal Road, Bakrol, Anand, Gujarat 388 315 India

**Keywords:** Pyrene, Sodium succinate, Heavy metals, Phthalic acid, Bioremediation, Enrichment technique

## Abstract

Polycyclic aromatic hydrocarbons (PAHs) are highly recalcitrant compounds due to their high hydrophobicity and tendency to partition in organic phase of soils. Pyrene is a high-molecular weight PAH, which has human health concerns. In the present study, a bacterial consortium, PBR, was developed from a long-term polluted site, viz., Amlakhadi, Ankleshwar, Gujarat, for effective degradation of pyrene. The consortium effectively metabolized pyrene as a sole source of carbon and energy. The consortium comprised three bacterial species, *Pseudomonas* sp. ASDP1, *Burkholderia* sp. ASDP2, and *Rhodococcus* sp. ASDP3. The maximum growth rate of consortium was 0.060/h and the maximum pyrene degradation rate was 16 mg/l/day. The organic and inorganic nutrients along with different surfactants did not affect pyrene degradation, but degradation rate moderately increased in the presence of sodium succinate. The significant characteristic of the consortium was that it possessed an ability to degrade six other hydrocarbons, both independently and simultaneously at 37 °C, in BHM (pH 7.0) under shaking conditions (150 rpm) and it showed resistance towards mercury at 10 mM concentration. Phthalic acid as one of the intermediates during pyrene degradation was detected through high-performance liquid chromatography (HPLC). The efficiency of consortium for pyrene degradation was validated in simulated microcosms’ study, which indicated that 99% of pyrene was metabolized by the consortium under ambient conditions.

**Electronic supplementary material:**

The online version of this article (doi:10.1007/s13205-017-0598-8) contains supplementary material, which is available to authorized users.

## Introduction

Polycyclic aromatic hydrocarbons (PAHs) are ubiquitous compounds present in the environment. Their origin in the environment may be attributed to extensive anthropogenic activities like industrialization, incomplete combustion of fossil fuels and oil spills or natural phenomena like wood fires, volcano eruptions and natural underwater oil spills **(**Juhasz and Naidu 2000; Atlas and Hazen 2011). PAHs are considered as priority pollutants by the US Environmental Protection Agency (USEPA). They persist in the environment for a long time because of the sequestration in sediment particles’ micropores and thus remain out of reach of microbial degradation. They are also concerned with human health because many PAHs are mutagenic, carcinogenic and teratogenic (Haritash and Kaushik 2009; Kumar et al. 2011).

Pyrene is high-molecular weight (HMW) PAH having very high hydrophobicity and because of its high octanol:water partition coefficient, there are relatively less reports of efficient degradation of this compound by microorganisms, though many efforts have been made by several investigators. In addition, few studies have reported on the utilization of these compounds as a sole carbon source (Bacosa and Inoue 2015; Ghosh et al. 2014; Ho et al. 2000; Wang et al. 2008). There are reports of degradation of pyrene by pure cultures and consortia developed from various origins like petroleum sludge, oil spill sites or other petroleum products contaminated sites. The isolated organisms may be from the Actinomycetes phylum (*Mycobacterium* sp.) or Proteobacteria (*Pseudomonas* sp., *Burkholderia* sp., etc.). But these are not very efficient degraders of HMW PAHs like pyrene. Bacosa et al. (2013) developed six consortia from sediment mainly consisting of different species of *Pseudomonas* and *Burkholderia* capable of degrading various PAHs. Availability of extra carbon or nitrogen sources may be limiting factor or the presence of toxic compounds may retard the degradation. So it is very useful to develop a versatile consortium that can degrade pyrene in the presence of other pollutants with their simultaneous degradation, since Amlakhadi canal received mixed pollutants from different surrounding industries (Patel et al. 2012a; Kathuria 2007).

The present study is focused on the development of efficient microbial consortium from the long-term polluted soil sediments of the Amlakhadi canal, Ankleshwar, which is the tributary of the Narmada River and polluted by extensive discharge of effluents from industries of the Ankleshwar Industrial Estate. Optimization of physicochemical parameters, effects of other additives and surfactants and effect of other hydrocarbons and heavy metals were other significant objectives. The most important objective of the study is a simultaneous degradation of mixture of PAHs (fluoranthene, pyrene, naphthalene, chrysene and phenanthrene) and related hydrocarbons (benzene, toluene and xylene) by developed consortium in simulated microcosms. Thus, study provides an important pace for the further bioremediation process like macrocosm study or reactor scale study.

## Materials and methods

### Media and chemicals

Bushnell-Haas broth (BHB), nutrient broth and nutrient agar were purchased from Himedia (Mumbai, India). All chemicals used in the study were of analytical and HPLC grade. Pyrene was purchased from Sigma-Aldrich^®^ (Bellefonte, Pennsylvania, USA) with 99% purity.

### Development of microbial consortium PBR for degradation of test PAHs

Polluted sediment samples were collected from Amlakhadi canal, Ankleshwar, Gujarat. The microbial consortium for the degradation of pyrene was developed by the culture enrichment method. Ten grams of sediment samples inoculated in 200 ml BHB medium amended with 1000 ppm of pyrene (from a stock solution of 50,000 ppm, filter sterilized by 0.2 µm nylon filters) and incubated under shaking conditions (150 rpm) at 37 °C for nearly 15 days. After 15 days the content of the flask was centrifuged at 200×*g* for 3 min to discard the sample debris. Ten to fifteen milliliters supernatant was inoculated into the fresh BHB amended with 1000 ppm of pyrene and continued incubation under shaking conditions 37 °C for another 15 days.

After subsequent incubation of 15 days the content of flasks was again centrifuged at 11,000×*g* rpm for 5 min to harvest the cells. The cells were re-suspended in minimal quantity of sterile distilled water and inoculated in fresh BHB containing 1000 ppm of pyrene. Thus, after repeated sub-culturing of more than 25 times in minimal medium without any nutritional additives and pyrene provided as sole carbon source, consistent degradation of pyrene with stable growth of the consortium was observed. Further, the degradation of pyrene was observed on Bushnell-Haas agar medium spread with 50 µl of stock solution of pyrene. Different dilutions of the consortium were spread on the solid media spiked with pyrene. After incubation of 15 days at 37 °C, Bushnell-Haas agar plates were checked for degradation of pyrene by observing zone of clearance around the colonies. The grown organisms were purified by streaking and regular transfers on solid agar plates.

### Characterization of microbial consortium

The enriched developed consortium was serially diluted and spread on Nutrient agar, Bushnell-Haas agar, Bushnell-Haas agar amended with 1000 ppm of pyrene and incubated for 1–8 days at various temperatures. Discrete colonies with distinctive morphology were further screened to pure cultures to enumerate the bacteria present in the consortium. Identification of bacterial isolates was performed using extracting genomic DNA from each pure culture using standard protocol of (Ausubel et al. 1997). 16S rRNA gene was amplified by eubacterial universal primers 8F and 1492R as described in (Desai and Madamwar 2007). The purified amplified product was sequenced using automated ABI 3500 Genetic Analyzer (Thermo Scientific, ABI, USA). Full length of 16S rRNA gene was analyzed by the BLAST tool at NCBI server to identify the bacteria.

### Development of Inoculum, degradation conditions and preparation of samples for HPLC

Consortium PBR was grown in BHB amended with 1000 ppm of pyrene under shaking conditions (150 rpm) at 37 °C. Five percent (v/v) of this grown consortium was used as inoculum for further studies. The detailed method has been provided in the Supplementary Information.

The pyrene degradation experiments were conducted in BHM medium amended with 100 ppm of pyrene under shaking conditions of 150 rpm at 37 °C. The PAHs degradation efficiency of consortium PBR was studied by extracting the entire content (100 ml) of the flask and its degraded products in 20 ml of dichloromethane by incubating the mixture under shaking condition at 150 rpm for 1 h followed by separation of aqueous and organic phases under static condition. The 500 µl of separated organic phase (i.e. dichloromethane) collected in fresh microfuge and the solvent was evaporated under vacuum using SpeedVac (Thermo Electron Corporation, Waltham, MA) and the dried content was re-suspended in 1 ml 70% acetonitrile. This prepared sample also diluted by 70% acetonitrile as the pyrene concentration will be in range of 10 ± 5 ppm to compare it with standard of 10 ppm pyrene. HPLC analysis was performed using Prominence LC system (Shimadzu, Japan), on Pursuit 3 PAH C_18_ reverse phase column (100 mm × 4.6 mm, 3 µm) (Agilent, USA), under ambient conditions, with acetonitrile:water (70:30, v/v) as eluent and isocratic flow rate of 1 ml/min. The standards and degraded products were detected at 254 nm with a Photo Diode Array Detector.

### Study of auxiliary nutrient and environmental parameters

For the effective degradation of pyrene by consortium PBR, several parameters were studied and optimized.

#### Effect of supplementary co-substrates

For enhancing the degradation potential of consortium PBR, BHM was supplemented with peptone, yeast extract, sodium succinate, ammonium nitrate (0.1%, w/v) or glucose (2.0%, w/v) and intermediates such as phthalic acid and salicylic acid (20 g/L) along with 100 ppm of pyrene. Uninoculated media with the respective co-substrates amended with 100 ppm of pyrene were served as abiotic controls. Another set of control experiments was performed with only BHB amended with 100 ppm of pyrene, inoculated with 5% of consortium PBR. All experiments were conducted under shaking conditions of 150 rpm at 37 °C, pH of 7.0 (unless specified) in triplicates.

#### Effect of environmental conditions

Different environmental factors viz. pH (5.0–9.0), temperature (30–50 °C), dissolved oxygen concentration with respect to shaking speed [0 (static), 50, 100 and 150 rpm], initial pyrene concentrations (100–4000 ppm), presence of different hydrocarbons [fluoranthene, phenanthrene and naphthalene (100 ppm)] other aromatic compounds [benzene, toluene and xylene (0.1%, w/v)], heavy metals at different concentrations [mercury (Hg), lead (Pb), chromium (Cr), cadmium (Cd) and zinc (Zn) at 1, 5 and 10 mM concentrations] surfactants CTAB (Cetyl-trimethyl ammonium bromide), SDS (sodium dodecyl sulfate), Tween-80 and Triton-X-100 (0.02% w/v and v/v) were studied to observed the variable effect on pyrene degradation by the consortium PBR.

The *µ*_max_, *K*_*S*_ and *q*_max_ were obtained from the exponential growth phase and degradation rate (while studying the initial substrate concentration) using the Monod equation as described in Ghosh et al. (2014) and Okpokwasili and Nweke (2005).

### Microcosm study

Microcosm study was performed to assess the potential of the developed consortium PBR for PAHs degradation in soil systems. In the study, microcosm experiments were modified from the method of Pathak et al. (2009, 2011). Since the soil sediments at Amlakhadi canal are completely submerged into the water, so as to mimic the natural environment, 50 g of pollutes and non-polluted soil samples were added in 100 ml of sterile water supplemented with BHM and inoculated with 5 × 10^7^ cells from exponential phase consortium PBR. Twelve different sets of experiments were conducted in triplicates as mentioned in Table 1. The experiments were performed for seven days, samples were withdrawn at third and seventh day, where 100 ml content was extracted in 20 ml of n-hexane and was mixed with 20 ml n-hexane and agitated on shaker at 120 rpm for 15 min. The mixture was allowed to settle further 10–20 min and nearly whole content (20 ml) of *n*-hexane was collected in fresh tube. The content was diluted to being the metabolite concentration in the of 10 ppm and degradation was measured spectrophotometrically against the blank of n-hexane using Double Beam Specord^®^ 210 BU UV–vis spectrophotometer (Analytica Jena AG, Germany) in spectral range of 190–500 nm.

**Table 1:** The effect on indigenous microflora and the ability of the consortium on pyrene degradation during microcosm studies

Experimental sets	Experimental parameters	Degradation (%)
Set 1	Pristine, non-sterile soil amended with 100 ppm pyrene, 100 ppm fluoranthene, 500 ppm naphthalene, 250 ppm phenanthrene and 5 ppm chrysene and consortium PBR	85
Set 2	Pristine, non-sterile soil amended with 100 ppm pyrene and consortium PBR	99
Set 3	Pristine, non-sterile soil amended with 100 ppm pyrene, to determine the ability of indigenous microflora for pyrene degradation	39
Set 4	Polluted, non-sterile soil amended with 100 ppm pyrene, 100 ppm fluoranthene, 500 ppm naphthalene, 250 ppm phenanthrene and 5 ppm chrysene and consortium PBR	99
Set 5	Polluted, non-sterile soil amended with 100 ppm pyrene, and consortium PBR	99
Set 6	Polluted, non-sterile soil amended with 100 ppm pyrene, to determine the ability of indigenous microflora for pyrene degradation	48
Set 7	Pristine, sterile soil amended with 100 ppm pyrene, 100 ppm fluoranthene, 500 ppm naphthalene, 250 ppm phenanthrene and 5 ppm chrysene and consortium PBR	86
Set 8	Pristine, sterile soil amended with 100 ppm pyrene and consortium PBR	77
Set 9	Pristine, sterile soil amended with 100 ppm pyrene, to determine abiotic loss of pyrene	3
Set 10	Polluted, sterile soil amended with 100 ppm pyrene, 100 ppm fluoranthene, 500 ppm naphthalene, 250 ppm phenanthrene and 5 ppm chrysene and consortium PBR	85
Set 11	Polluted, sterile soil amended with 100 ppm pyrene, and consortium PBR	75
Set 12	Polluted, sterile soil amended with 100 ppm pyrene, to determine abiotic loss	1

## Results and discussion

Pyrene is a high-molecular weight polycyclic aromatic hydrocarbon having low water solubility (0.12–0.18 mg/L) which, therefore, tends to sequester in the sediments. Hence, there are relatively very less reports on efficient microbial degradation of pyrene. The present study demonstrated the development of consortium PBR (and its characterization) for the degradation of pyrene from the polluted sediments of Amlakhadi canal, Ankleshwar, Gujarat. The physicochemical parameters of Amlakhadi are shown in Table S1. From Table S1 it is clear that there is the presence of heavy metals in the sediments of the Amlakhadi canal. More than 1200 industrial units are manufacturing petroleum products, chemicals, pesticides, pharmaceuticals, bulk drugs, engineering, plastic and many other products and effluents from these industries are released into the Amlakhadi canal. The continual release of these effluents (for the last four decades) containing compounds of xenobiotic origin, have acclimatize the inhabitant microorganisms which have evolved the necessary resilient mechanisms for their metabolism. Hence it was a pertinent site to obtain the enriched bacterial community, having the inherent capacity to metabolize PAHs.

### Development of bacterial consortium PBR and bacterial identification

**Fig. 1:**
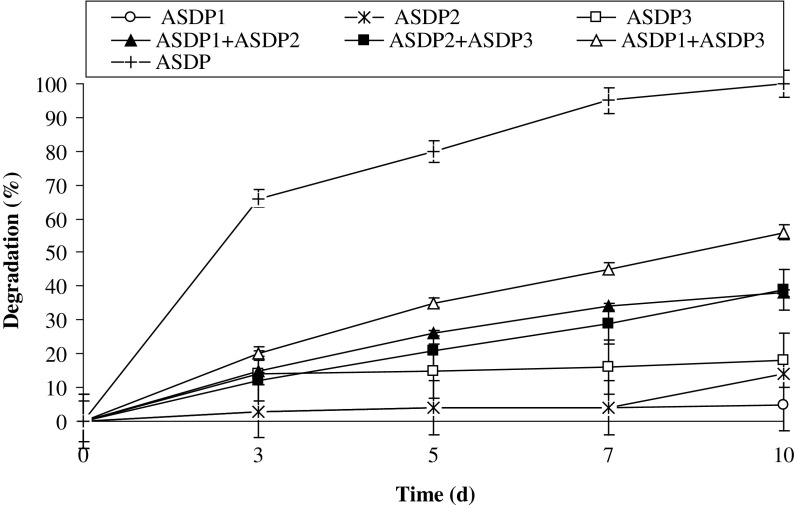
Degradation of pyrene (100 ppm) by individual constituent pure cultures, combination of the pure cultures and consortium under optimized conditions (37 °C and pH 7, 150 rpm) in BHM. ASDP1: *Pseudomonas* sp. ASDP1, ASDP2: *Burkholderia* sp. ASDP2, ASDP3: *Rhodococcus* sp. ASDP3, PBR: Consortium

The consortium PBR having pyrene catabolizing ability was developed through culture enrichment method in Bushnell-Haas medium amended with pyrene using sediments of Amlakhadi canal. With each consecutive transfer of the consortium, pyrene degradation efficiency has concurrently enhanced and stable growth of the constituent bacterial species was also observed. During the process of consortium enrichment, samples were intermittently withdrawn to assess the degradation efficiency and were simultaneously spread on different media as mentioned above to screen bacteria constituting the consortium. The distinct bacterial colonies with unique morphology were further purified and identified as *Pseudomonas* sp. ASDP1 (NCBI Accession No.: KU375114), *Burkholderia* sp. ASDP2 (KU375115) and *Rhodococcus* sp. ASDP3 (KU375116). All the three bacteria are well known for pyrene degradation (Juhasz and Naidu 2000). Figure 1 shows the degradation profile of each constituent individual culture of the consortium. It was evidently observed the incompetence of the individual bacterial species in pyrene degradation, where even after ten days 5, 14 and 18% of initial pyrene concentration was degraded by ASDP1, ASDP2 and ASDP3, respectively. However, nearly complete degradation of 100 ppm of pyrene was observed within seven days by the consortium PBR comprising of above three species. Therefore, co-metabolism may play pivotal role in degradation of pyrene.

### Growth rate and effect of substrate concentration

It is imperative to determine the load of a pollutant that can be degraded by defined number of microorganisms. In a polluted environment, there is a range of concentrations of different pollutants and microorganisms have different sensitivities towards the various concentrations of pollutants (Leahy and Colwell 1990; Boopathy 2000). In kinetic terms, there is always a threshold concentration below which the pollutants cannot be detected by particular numbers of organisms and above a particular concentration of pollutant; it will prove toxic to microorganisms (Doick et al. 2005; Okpokwasili and Nweke 2005 **)**. Moreover, it was generally observed that in certain confined range, as the concentration of pollutant increases, the rate of degradation of that pollutant also increases. This can be determined by kinetic parameters viz. specific growth rate (*µ*), specific degradation rate (*q*) and half saturation rate constant (*k*).

**Fig. 2:**
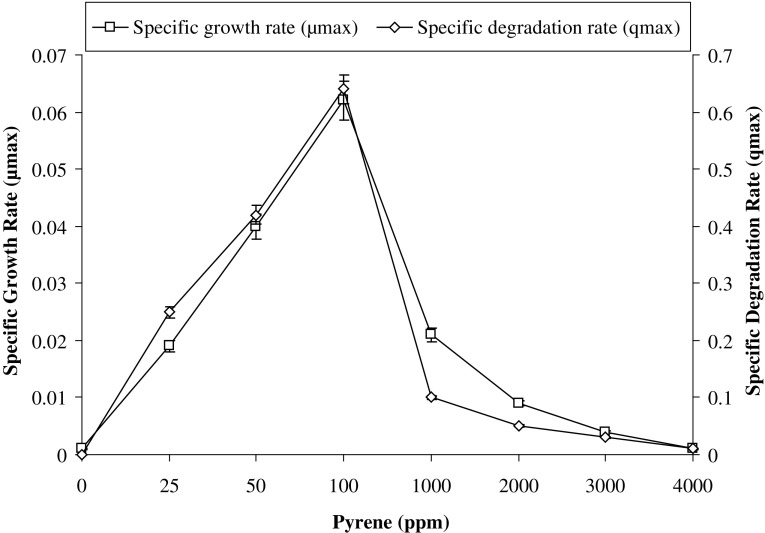
Effect of pyrene concentrations on its degradation rate (*q*_max_) and on growth rate (*μ*_max_) of the consortium at 37 °C and pH 7, 150 rpm in BHM

From Fig. 2, it was clear that 100 ppm of pyrene was efficiently degraded within 7 days as compared to higher concentrations. The degradation efficiency of PBR gradually decreases with increasing pyrene concentrations and it falls nearly 16-fold, where only 640 ppm (i.e. 16%) of 4000 ppm pyrene was degraded by the consortium after 7 days. The results from Fig. 2 further revealed that, the specific degradation rate (*q*) and specific growth rate (*µ*), increases linearly with the increase in pyrene concentrations, in lower range of 25–100 ppm. However, both these rates subsequently decreased at higher concentrations from 1000 to 4000 ppm. Maximum growth rate (*µ*_max_), half saturation coefficient (*K*_*S*_) and maximum degradation rate (*q*_max_) of pyrene for consortium PBR were 0.062/h, 6 mg/L and 14 mg/L/d, respectively. With the increase in pyrene concentration, the sharp fall in degradation as well as growth rates was observed (Fig. 2). The growth rate decreased nearly 3-fold at 1000 ppm (0.021/h) and 62-fold at 4000 ppm (0.001/h). Whereas the degradation rate correspondingly deceased nearly 3-fold at 1000 ppm (5.1 mg/L/d) and 64-fold at 4000 ppm (0.25 mg/L/d). Therefore, it could be assessed that, the degradation of pyrene was dependent on the growth of the consortium and it evidently suggested that the decrease in pyrene concentration is biologically mediated rather than abiotic loss. Further, the higher concentrations of pyrene were inhibitory and might be toxic to the consortium retarding the growth of the bacteria.

Thus, the results provided the significant implications about the substrate sensitivity of the consortium PBR, which prompted us to conduct further experimentation at 100 ppm of pyrene. It was observed that, the efficiency of xenobiotic degradation by microorganisms mostly dependent on the environmental conditions and different parameters needs to be studied precisely so as it can be applied further for large scale actual onsite or reactor scale remediation process. Hence, the effect of each significant factor for PAHs degradation was studied to enhance the degradation potential of the consortium PBR.

### Effect of temperature, pH and oxygen concentration

**Fig. 3:**
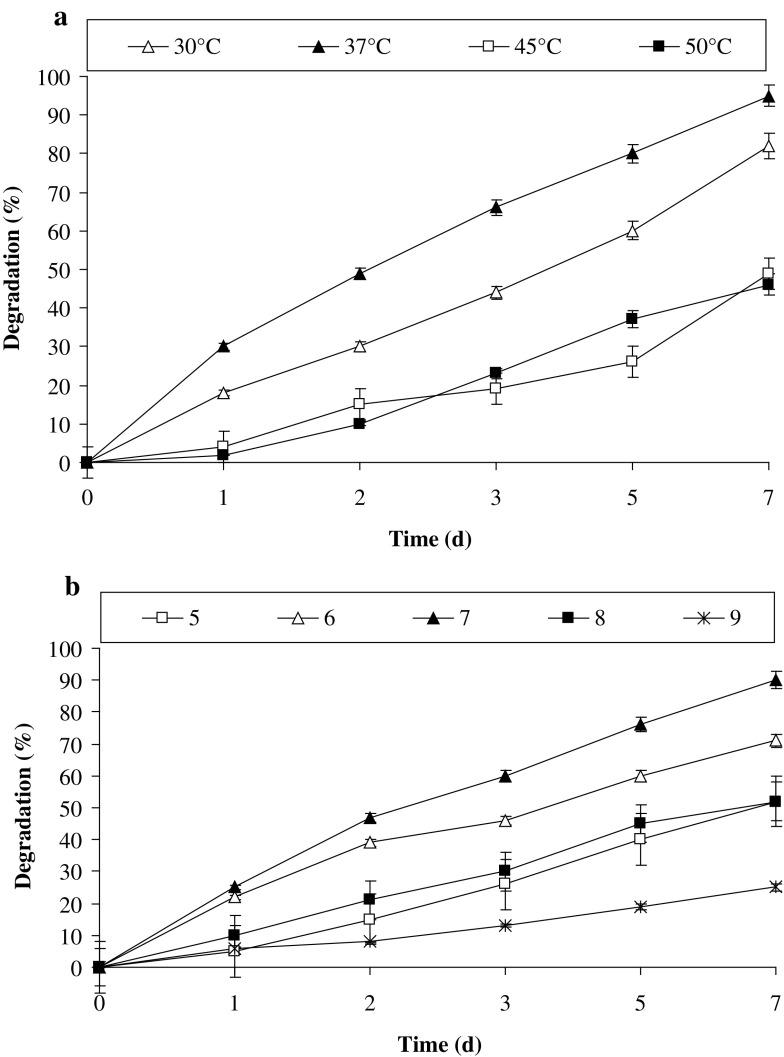
Effect of **a** temperature and **b** pH on the degradation of pyrene by consortium PBR at 150 rpm in BHM

Temperature is one of the important environmental and abiotic parameters. Biodegradation is also a chemical reaction, and enzymatic catalysis carried out by microorganisms generally lies in the optimum (growth) temperature range. Amlakhadi canal and its surrounding geographical region have relatively temperate climate with temperature ranging from 15 to 45 °C (±2 °C) was commonly observed during winter to summer, respectively. Therefore, the degradation experiments were performed between 30 and 50 °C. The results in Fig. 3a suggest that a lower temperature is more favorable for optimum degradation of pyrene. At 37 °C about 80% of 100 ppm of pyrene was effectively degraded; however, the maximum degradation (95%) by PBR could be obtained at 37 °C after 7 days. The results revealed the decreases in the degradation rate at higher temperature and the degradation rate nearly declined by 2.1-fold at 50 °C. Nearly all aerobic enzymes for the degradation of PAHs work optimally in the range 30–37 °C (Patel et al. 2012a, b; Hamzah et al. 2011; Mukherjee and Roy 2013).

pH is another important factor in the biochemical reactions carried out by enzymes that catalyze the reactions. Figure 3b demonstrates the effect of pH on the degradation of pyrene by PBR. It suggested that for better/efficient degradation of pyrene, lower pH was more effective. At pH 6.0, 70% of 100 ppm of pyrene was degraded within 7 days, while the maximum degradation (97%) was observed at pH 7.0. At higher pH 9.0 nearly 1.8-fold decrease in degradation rate was observed. In various earlier studies it was observed that setting of pH to near neutrality has proven beneficial for the bioremediation of gasoline-contaminated soil, oily sludge in the soil and degradation of octadecane, naphthalene, and other PAHs, e.g. pyrene (Leahy and Colwell 1990; Verstraete et al. 1976; Patrick and DeLaune 1977; Hambrick et al. 1980; Dibble and Bartha 1979). In a recent work, Ravanipour et al. (2015) proved that pH 6.8 was better for the development and maintenance of bacterial consortia for the phenanthrene degradation in artificially contaminated soil. Pure culture study of degradation of pyrene by *Rhodococcus* sp. UW1 also indicated that, maximum degradation of pyrene and highest activity of dioxygenase system was found at pH 7.1 and 7.2, respectively (Walter et al. 1991).

**Fig. 4:**
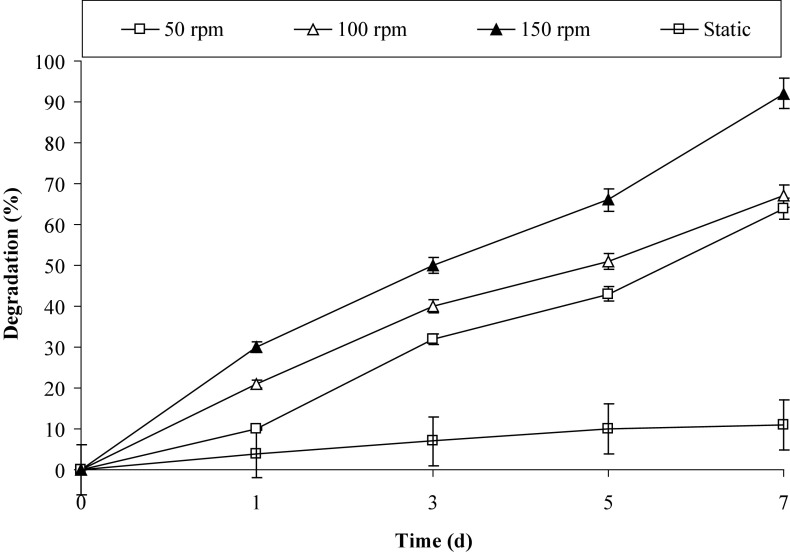
Effect of various shaking and static conditions on the degradation of pyrene by consortium PBR at 37 °C in BHM, pH 7.0

The multi-component monooxygenases and dioxygenases playing a significant role in the initial steps of PAHs degradation essentially required molecular oxygen (Leahy and Colwell 1990). Besides, oxygen is a primary requirement for the aerobic degradation of nutrients. The results from Fig. 4 support the above notion and reveal that environments, which lack molecular oxygen, did not support pyrene degradation, i.e. only 11% of pyrene was degraded by PBR after 7 days under static conditions. But as soon as, the oxygen concentration increases (i.e. at 50/100 rpm of shaking) degradation rate enhanced by 6-fold; however, maximum degradation was observed under 150 rpm with increase in 9-fold in metabolic activity of PBR for pyrene degradation. Moreover, with the increase in the shaking speed, the opportunity of contact between bacterial cells and PAHs also increases, which might enhance the rate of degradation.

Nevertheless, PAHs with 2–3 rings are observed to be degraded under anaerobic conditions at very slow rates, but there are very rare observations for the degradation of PAHs with more than three rings (Johnson et al. 2005). Proportional utilization of PAH carbon to natural organic carbon is 3 orders of magnitude higher during cooler months when water temperatures are low and dissolved oxygen (DO) percent saturation is higher and infusion of cooler, well-oxygenated water to the water column overlying contaminated sediments during summer stimulates PAH metabolism (Boyd et al. 2005). In the context of these observations, it was obvious that degradation of pyrene, which possesses four ring clusters, requires high dissolved oxygen concentration and in turn higher shaking conditions.

### Effect of organic carbon sources, intermediates and surfactants

**Fig. 5:**
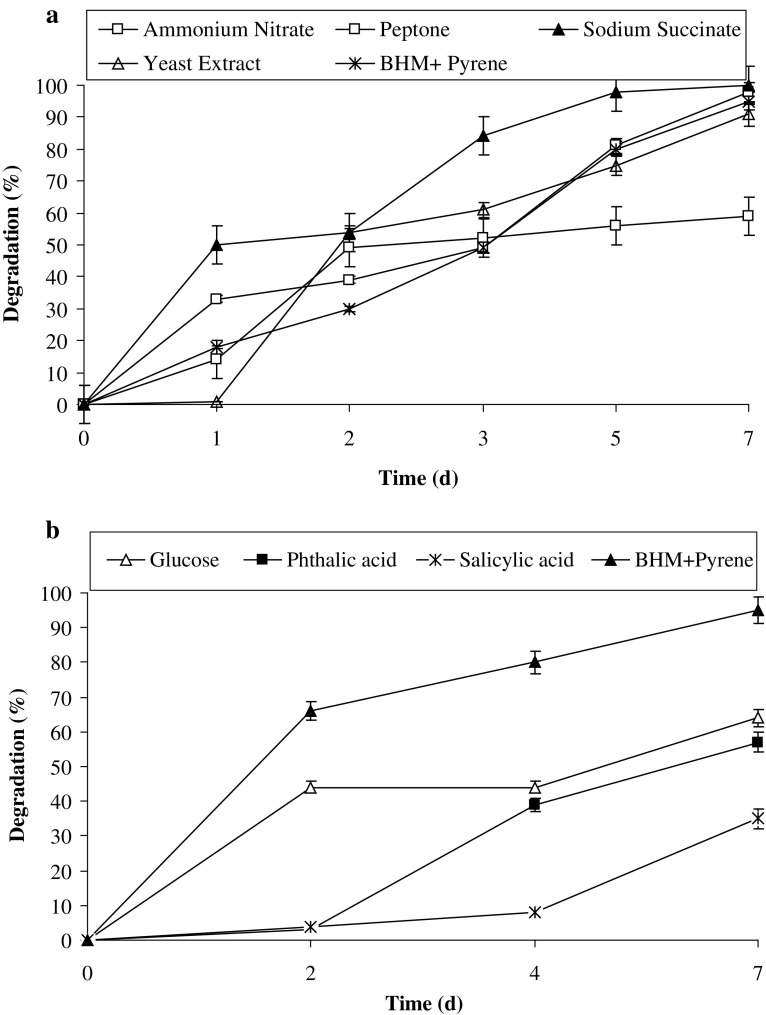
Effect of **a** various organic and inorganic sources and **b** glucose and intermediates on pyrene degradation by the consortium PBR under optimized conditions (37 °C and pH 7, 150 rpm) in BHM

The availability of electron rich reduced substrates in the environment and the accessibility of PAHs to microorganisms (owing to their highly hydrophobic nature) are few significant factors that affect the degradation of these compounds. Figure 5a, b shows the effect of different compounds on pyrene degradation and it can be observed that both organic sources, peptone and yeast extract had negligible effect on pyrene degradation, while the rate of degradation decreased by 1.5- and 1.6-fold in the presence of glucose and ammonium nitrate, respectively. Similarly, upon providing phthalic acid and salicylic acid, the end products of the pyrene degradation (Seo et al. 2009), the degradation rate also decreased by 1.7- and 2.75-fold, respectively. It seems that both these compounds may inhibit the degradation pathway by feedback inhibition of degradation enzymes. The study further revealed the interesting results; it was observed that on providing sodium succinate (Fig. 5a), degradation rate was enhanced by 1.7-fold and complete degradation of 100 ppm of pyrene was obtained within 5 days. The positive effect of succinate could be related to the TCA cycle intermediate, which may provide direct utilization of the compound bypassing the rate-limiting steps in the pathway.

**Fig. 6:**
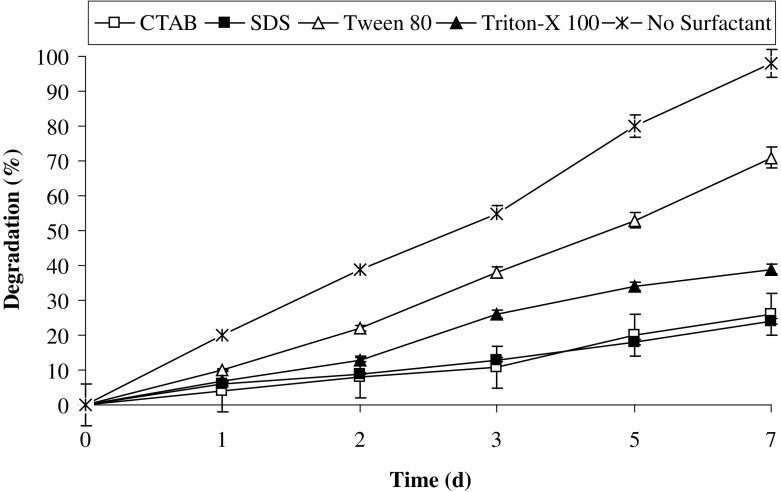
Effect of different surfactants on pyrene degradation by the consortium PBR under optimized conditions (37 °C and pH 7, 150 rpm) in BHM

In the natural environment PAHs tend to sequester in sediments where they are out of reach of microbial degradation (Boopathy 2000). Their availability to the microorganism can be increased by supplementing the surfactants. The observed results showed that all the surfactants used in the study did not enhance degradation potential of the consortium. However, their supplementation decreased the pyrene degradation rate. Figure 6 revealed a 3.8-fold decrease in the presence of CTAB, while the degradation rate was decreased by 4.1-, 1.4-, and 2.5-fold in the presence of SDS, Tween 80, and Triton-X100. The decrease in the degradation rate of pyrene by consortium on supplementing the surfactants is mainly due to preferential utilization of surfactant as carbon and energy source over pyrene. Few of the surfactants would be toxic to the consortium which has resulted in the decrease in the pyrene degradation (Shuttleworth and Cerniglia 1995; Mrozik et al. 2003; Pathak et al. 2009).

In our earlier studies, similar observations were made, where naphthalene degradation by *Pseudomonas* sp. HOB1 was not enhanced by addition of external surfactants (Pathak et al. 2009). In another study, Patel et al. (2012) also observed that external supplementation of surfactants did not enhance the degradation of phenanthrene by *Pseudoxanthomonas* sp. DMVP2. The anionic and cationic surfactants SDS and CTAB, respectively, decreased the phenanthrene degradation by consortium ASP (Patel and Madamwar 2013). These results additionally suggested that the strains involved in PAHs degradation might be producing bio-surfactant and further supplementation of surfactant may have negative effect on PAHs degradation (Pathak et al. 2009).

### Effect of inoculum size

**Fig. 7:**
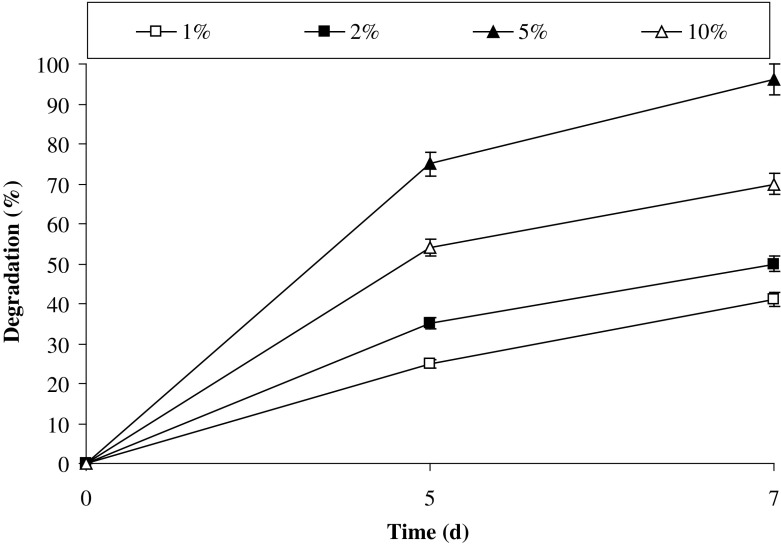
Effect of inoculum size on pyrene degradation by the consortium PBR under optimized conditions (37 °C and pH 7, 150 rpm) in BHM

Though consortia may contain different PAH degrading bacteria, there is always one threshold of a number of organisms above which degradation occurs optimally and below which the rate of degradation is quite low (Okpokwasili and Nweke 2005). If the inoculum size was increased above the optimal level, there was no increase in degradation rate of pollutant as at greater inoculum size the organisms may rapidly reach in stationary phase of growth curve of microorganisms (Patel et al. 2012a; Abdelhay et al. 2008). In supporting the above notion, the results from Fig. 7 demonstrated that 5% (v/v) inoculum was the optimum inoculum size for efficient degradation of pyrene. Below and above 5% inoculum size the rate of degradation decreased, the similar results were observed by Abdelhay et al. (2008).

### Effect of other hydrocarbons on pyrene degradation and simultaneous degradation of different PAHs

**Fig. 8:**
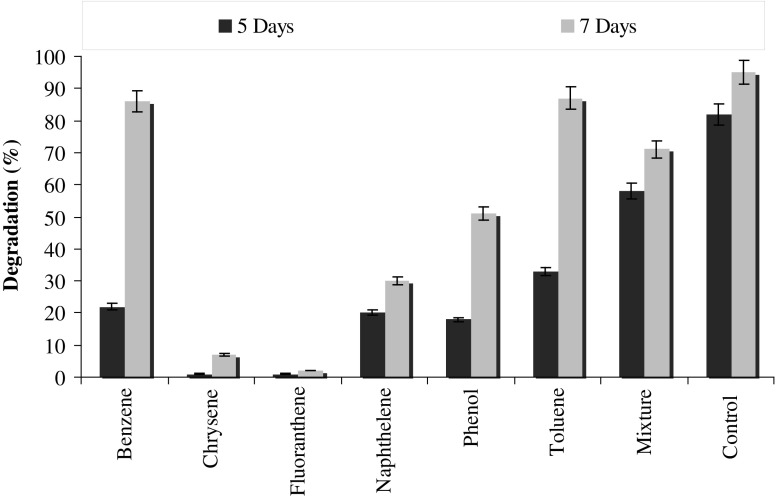
Effect of different hydrocarbons on pyrene degradation by the consortium PBR under optimized conditions (37 °C and pH 7, 150 rpm) in BHM

Polluted ecosystems always consist of various mixtures of compounds in different concentrations. It is always important to assess the degradation potential of the consortium for their individual and simultaneous degradation of different pollutants. Though, simultaneous degradation of different compounds is a different issue from this where the developed consortia uses multiple strategies to degrade different compounds simultaneously. Therefore, a study has been designed to assess the potential of the consortium PBR for simultaneous degradation of the different PAHs and the effect of other hydrocarbons on the degradation of pyrene. It can be observed from Fig. 8 that chrysene and fluoranthene completely inhibited pyrene degradation, while it was decreased by 3.1-fold in the presence of naphthalene. The results further revealed that nearly 85% of initial concentration of the pyrene was degraded in the presence of benzene and toluene. Whereas on providing six different hydrocarbons together, 71% pyrene was degraded (Fig. 8). The study by Bacosa and Inoue (2015) showed that different consortia have different potentials for using PAHs as the sole carbon source. Their study also revealed that few consortia can degrade pyrene as the sole source of carbon. But other consortia required earlier exposure of PAHs for metabolism of pyrene.

It is the most important and positive aspect of the developed consortium because during further studies like microcosm or reactor level the consortium must grow in presence of mixture of compounds. The decrease in the rate of degradation of pyrene in the presence of other hydrocarbons may be due to the toxicity of aromatic hydrocarbons like chrysene and fluoranthene. The toxicity is believed to be due to cell membrane disruption by aromatic hydrocarbons (Jacques et al. 2005). Moreover, due to structural similarity of many hydrocarbons, the bacterial oxygenases may utilize more than one hydrocarbon and co-metabolize different substrates, however, at significantly low rates (Ascon-Cabrera and Lebeault 1993). The observed results are very similar with this notion because degradation of pyrene occurs in the presence of other hydrocarbons but at very low rates relative to control experiment where no other hydrocarbons are added.

**Fig. 9:**
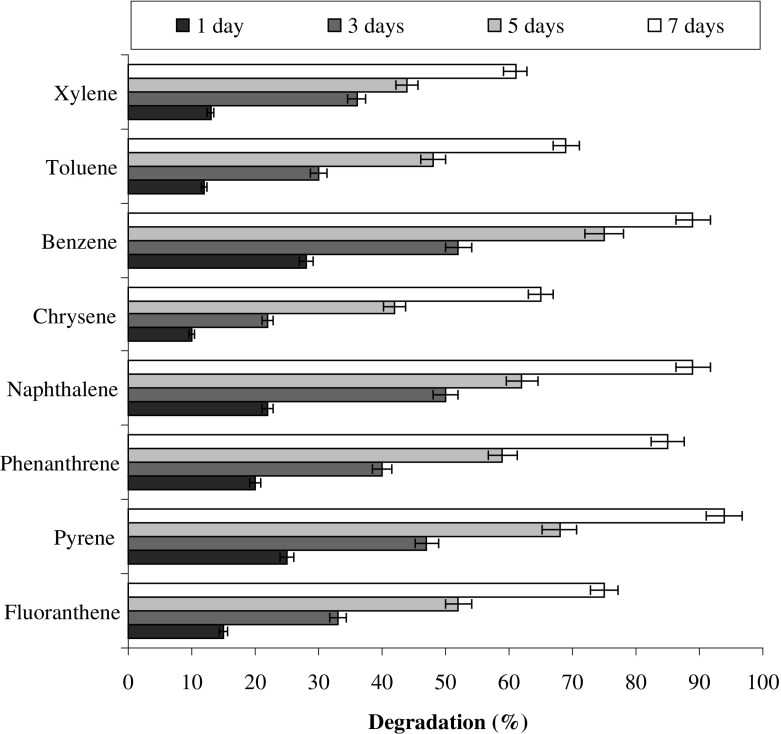
Simultaneous degradation of other hydrocarbons along with pyrene degradation by the consortium PBR under optimized conditions (37 °C and pH 7, 150 rpm) in BHM

Further, in a different study, the results from Fig. 9 revealed that above 60% of the all the PAHs (eight hydrocarbons) used in the experiments were degraded by the consortium. The degree of degradation of PAHs varies depending upon the test compound. Because the consortium was acclimatized with pyrene maximum degradation was observed with pyrene, followed by naphthalene (89%), benzene (89%) and phenanthrene (85%). The higher rate of degradation of the all the compounds can be attributed to the co-metabolism. Co-metabolism is a very important phenomenon in nature where multiple compounds having similar structures can be degraded concurrently. Yuan et al. (2002) describes that degradation efficiency of microorganisms was more vigorous when acenaphthene, fluorene, phenanthrene, anthracene, and pyrene are present simultaneously compared to the rate of degradation when the PAHs are present individually because the presence of all five compounds provides more carbon source, or cross-acclimation may enhance the rate of biodegradation.

### Effect of heavy metals

**Fig. 10:**
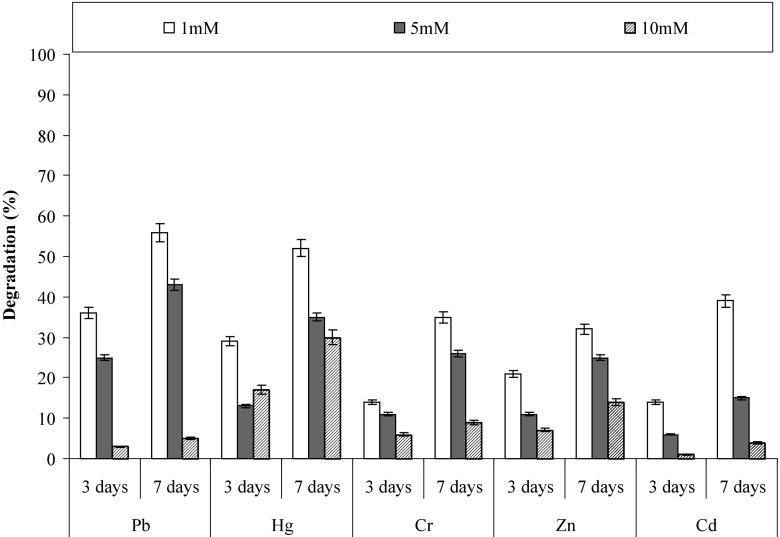
Effect of different heavy metals on pyrene degradation by the consortium PBR under optimized conditions (37 °C and pH 7, 150 rpm) in BHM

Heavy metals are one of the major characteristic of polluted ecosystems such as polluted water bodies, aquifers and polluted sediments. They are inhibitory to microbial enzymes essential for different metabolic reactions and therefore inhibitory to the microbial growth (Chen et al. 2008). The study was performed to assess the effect of five heavy metals (Pb^2+^, Hg^2+^, Cr^2+^, Zn^2+^ and Cd^2+^) at three different concentrations (1,5 and 10 mM) on the pyrene degradation. It is very apparent from Fig. 10 that pyrene degradation was affected in the presence of heavy metals and it significantly decreases at higher concentrations. The results revealed surprising observations, that pyrene degradation was affected severely in the presence of chromium, zinc and cadmium than lead and mercury. The degradation efficiency progressively decreased as the concentration of the heavy metals increased. Nearly 35% degradation was observed after seven days in the presence of chromium, zinc and cadmium at 1 mM concentration each, while at the same concentration about more than 50% degradation was observed in the presence of lead and mercury.

The study also revealed another significant observation that the consortium was more resistant towards mercury, i.e. at higher concentration (10 mM) nearly 30% pyrene was degraded after seven days, which was less than 10 mM for another four heavy metals under similar conditions. Moreover, Figure S1 shows the relationship between percent growth retardation by different concentrations of heavy metals which was measured as described by Pepi et al. 2008. EC_50_ values represent the 50% reduction in the growth of the consortium at the different concentrations of heavy metals. For the consortium of pyrene degradation the EC50 for Pb^2+^, Hg^2+^, Cr^2+^, Zn^2+^ and Cd^2+^ is 6, 5, 6.9, 5.2 and 5.4 mM, respectively. The high tolerance to heavy metals may be due to production of bio-surfactant by the consortium (Sandrin et al. 2000) or precipitation of heavy metals by phosphate and sulfate of the medium (Patel et al. 2012b; Hughes and Poole 1991). At higher concentrations lead, mercury and cadmium were precipitated and therefore may not retard the degradation of test PAHs.

### Degradation profile of pyrene and stoichiometry

**Fig. 11:**
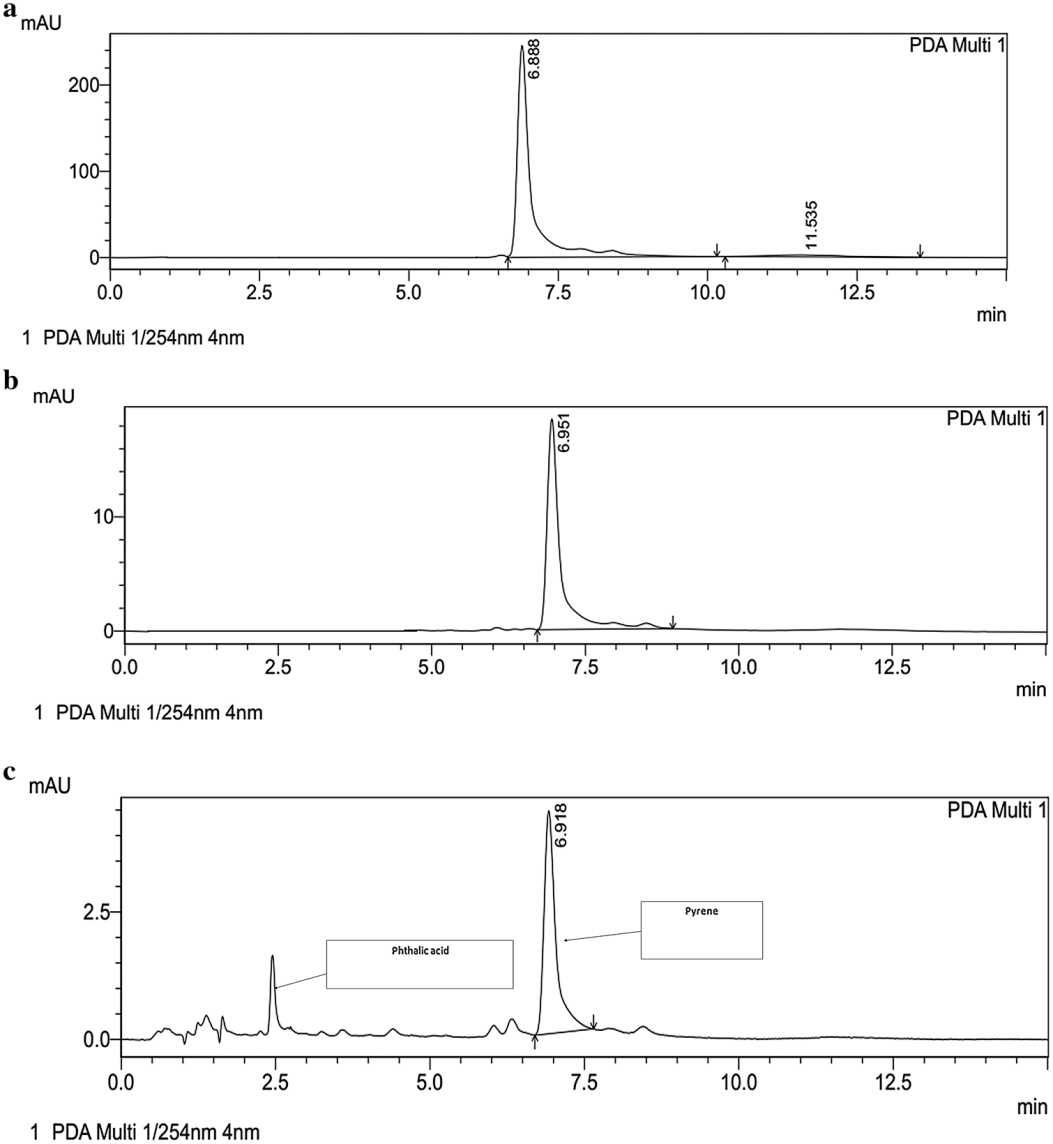
HPLC chromatogram of **a** intact pyrene, **b** degraded intermediates after 3 days and **c** degraded intermediates after 7 days

**Fig. 12:**
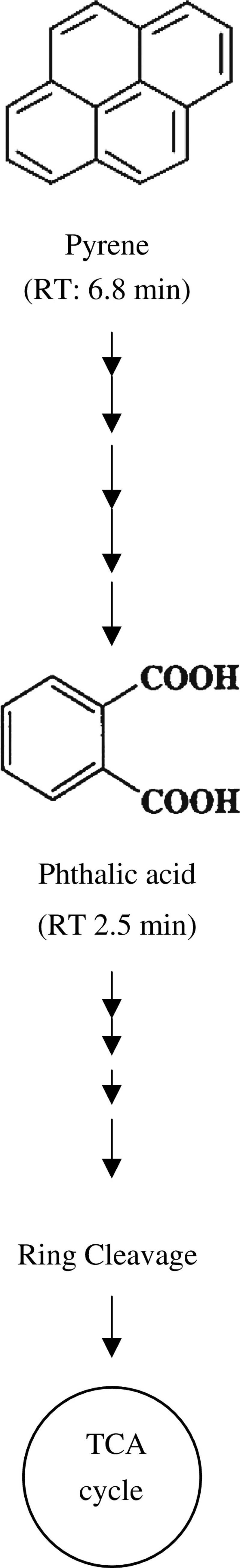
Proposed pathway for degradation of Pyrene by the consortium PBR under optimized conditions

The competence of the consortium for pyrene degradation and its degradation pattern was studied through HPLC. The chromatogram of intact pyrene showed a single major peak at retention time of 6.8 min (Fig. 11a). Degradation of pyrene by consortium PBR can be observed by the gradual decrease in the peak of pyrene from 250 mAU (0 day) to 3.5 mAU (7 days) at retention time near 6.8 min. The observed results evidently indicated the formation of phthalic acid, having the retention time of 2.5 min (Fig. 11b, c). Therefore, it can be postulated that pyrene was degraded through phthalic acid pathway by the consortium PBR and the proposed pathway is as depicted in Fig. 12.

The stoichiometric relation between pyrene and phthalic acid was established and the results indicated that, from 0.4 mM of pyrene, 0.003 mM of phthalic acid was produced through synergistic metabolism of consortial species. That is, the stoichiometric ratio of nearly 1:100 between pyrene and phthalic acid can be postulated for 100 ppm of initial pyrene concentration and can be correlated from HPLC chromatogram of degraded products of pyrene (Fig. 11a, c). Therefore, the results evidently suggested that phthalic acid is not a dead end product of bacterial consortial metabolism, because the ratio of pyrene:phthalic acid was at much lower side. If it could have been a dead end product, the expected ratio would have higher value. Moreover, from Fig. 11c, the HPLC chromatogram of pyrene degradation revealed the emergence of other peaks at different retention time, along with major peak of phthalic acid. The difference between peaks of native pyrene molecule and phthalic acid was comparatively higher which clearly suggested that phthalic acid was not accumulated in the medium and effectively being metabolized by consortium PBR.

This is an important observation for the complete mineralization of pyrene, as Krishnan et al. (2004) observed the accumulation of phthalic acid while monitoring the degradation of phenanthrene by *Pseudomonas* sp. strain PP2, which have similar structural properties with pyrene. This implied the importance of consortium, where two or more microorganisms synergistically degrade and metabolize the xenobiotic compounds which is recalcitrant for complete degradation by single organism.

### Microcosm studies

The microcosm studies provide an insightful observation about the competence of the consortium PBR for pyrene and other PAHs degradation under soil system and in the presence of the native microflora of polluted and pristine soils. Results from Table 1 evidently suggested that indigenous microflora of the polluted and pristine soils can degrade 39% of the pyrene without augmentation of consortium PBR whereas augmentation increases the degradation of pyrene in polluted and pristine soil to 99%. This indicates the ability of developed consortium to work exclusively in presence of components of pristine soil as well as polluted indigenous which reveal the high efficiency of PBR consortium and reveal positive effect of augmentation of PBR consortium. Also the abiotic loss of PAH is negligible in presence of sterile polluted and pristine soil. It can be noted here that in sterile polluted and pristine soils the degradation occurs at 75 and 77%, respectively, as compared to 99% degradation in both non-sterile polluted and pristine soils, which indicates the aid of indigenous microflora for the degradation by PBR consortium. Thus, indigenous microflora also works in cooperation with consortium to increase the degradation rate of pyrene. Also the degradation of pyrene was higher in the presence of non-sterile polluted samples as compared to sterile polluted samples. The λ_max_ for pyrene in 70% ACN was 340 nm and for degradation calculation the O.D. at this wavelength was taken into consideration. But due to the cyclic structure and multiple benzene rings, pyrene has a complex absorption spectrum in the UV region that is from 200 to 400 nm. Thus, consortium PBR is highly efficient in degrading pyrene in microcosm and may be applicable for the macrocosm and reactor scale study.

## Conclusion

The study revealed the effectiveness of developed consortium PBR for pyrene degradation, where it was metabolized as a sole source of carbon and energy through the phthalic acid pathway. The competence of the consortium was revealed by the observations that it individually and simultaneously degraded six different hydrocarbons other than pyrene without supplementing any additional nutrient in BHM. Owing to the growth of consortium and degradation of pyrene in presence of other PAHs and heavy metals, low requirements like nutritional additives and surfactants and successful working in simulated microcosms, the developed consortium is indeed an efficient consortium and can be further used for macrocosm and reactor scale studies.

## Electronic supplementary material

Below is the link to the electronic supplementary material.

Supplementary material 1 (DOC 39 kb)
