# Supplementary material for: Metabolism of pyrene through phthalic acid pathway by enriched bacterial consortium composed of Pseudomonas, Burkholderia, and Rhodococcus (PBR)
Source: 3 Biotech. 2017 Apr 11;7(1):29. doi: 10.1007/s13205-017-0598-8 (PMC5388654; doi:10.1007/s13205-017-0598-8)
Supplement: Supplementary file 1 — Supplementary material 1 (DOC 39 kb) [file 13205_2017_598_MOESM1_ESM.doc]

**Supplementary Information**

**Metabolism of pyrene through phthalic acid pathway by enriched bacterial consortium composed of *Pseudomonas*, *Burkholderia*, and *Rhodoccocus* (PBR)**

Sagar Vaidya, Kunal Jain, Datta Madamwar^*^

Environmental Genomics and Proteomics Lab, UGC Centre of Advanced Study, P.G. Department of Biosciences, Satellite Campus, Vadtal Road, Sardar Patel University, Bakrol – 388 315, Anand, Gujarat, India

***Corresponding author**

Tel.: +91 2692 229380

Fax: +91 2692 236475

Email addresses: [sagarvaidya207@gmail.com](mailto:sagarvaidya207@gmail.com) (S. Vaidya)

[datta_madamwar@yahoo.com](mailto:datta_madamwar@yahoo.com) (D. Madamwar)

**2.0 Materials and Methods**

**2.4 Development of Inoculum, degradation conditions and preparation of samples for HPLC**

Three different organisms which constituted the consortium ASDP were grown with 200 ppm of pyrene as sole source of carbon in a single 500 ml Erlenmeyer flask containing 200 ml of BHM. The consortium was allowed to grow till 15 days. The bacterial growth at log phase was collected by centrifugation (6000 x g) for 5 min and growth was washed with sterile deionised water three times and suspended in it (20 ml) as the absorbance at 600 nm was 1.0 which represented the bacterial number 1.0 x 10^7^ cells/ml determined by serial dilution plate counts. From this consortium 1%, 2%, 5% and 10% (v/v) inoculum was added in separate flasks containing 100 ml of BHM spiked with 200 ppm pyrene and determined which inoculum size was appropriate for efficient degradation of pyrene in 15 days.

**Table S1:** Physico-chemical characteristic of the soil sediments of Amlakhadi canal, Ankleshwar

| **Physicochemical parameter** | **Concentration (mg/kg)** |
| --- | --- |
| Iron | 1.323 |
| Copper | 0.912 |
| Nickel | 0.223 |
| Chromium | 0.321 |
| Manganese | 1.400 |
| Magnesium | 28.400 |
| Mercury | Below detection level |
| Cadmium | 0.004 |
| Lead | 0.240 |
| Zinc | 1.050 |
| Ammonical Nitrogen | 60.000 |

**Figure S1:** The effect of heavy metals on percent growth retardation of the consortium ASDP, during degradation of pyrene
